# Successful classification of cocaine dependence using brain imaging: a generalizable machine learning approach

**DOI:** 10.1186/s12859-016-1218-z

**Published:** 2016-10-06

**Authors:** Mutlu Mete, Unal Sakoglu, Jeffrey S. Spence, Michael D. Devous, Thomas S. Harris, Bryon Adinoff

**Affiliations:** 1 Department of Computer Science and Information Systems, Texas A&M University-Commerce, Commerce, TX USA; 2 Computer Engineering, University of Houston – Clear Lake, Houston, TX USA; 3 Center for Brain Health, University of Texas at Dallas, Richardson, TX USA; 4 Department of Neurology, UT Southwestern Medical Center, Dallas, TX USA; 5 Avid Radiopharmaceuticals, Philadelphia, PA USA; 6 Veterans Affairs North Texas Health Care System, Dallas, TX USA; 7 Department of Psychiatry, UT Southwestern Medical Center, Dallas, TX USA

**Keywords:** Substance use disorders, Cocaine dependence, Machine learning, Support vector machines, Classification

## Abstract

**Background:**

Neuroimaging studies have yielded significant advances in the understanding of neural processes relevant to the development and persistence of addiction. However, these advances have not explored extensively for diagnostic accuracy in human subjects. The aim of this study was to develop a statistical approach, using a machine learning framework, to correctly classify brain images of cocaine-dependent participants and healthy controls. In this study, a framework suitable for educing potential brain regions that differed between the two groups was developed and implemented. Single Photon Emission Computerized Tomography (SPECT) images obtained during rest or a saline infusion in three cohorts of 2–4 week abstinent cocaine-dependent participants (*n* = 93) and healthy controls (*n* = 69) were used to develop a classification model. An information theoretic-based feature selection algorithm was first conducted to reduce the number of voxels. A density-based clustering algorithm was then used to form spatially connected voxel clouds in three-dimensional space. A statistical classifier, Support Vectors Machine (SVM), was then used for participant classification. Statistically insignificant voxels of spatially connected brain regions were removed iteratively and classification accuracy was reported through the iterations.

**Results:**

The voxel-based analysis identified 1,500 spatially connected voxels in 30 distinct clusters after a grid search in SVM parameters. Participants were successfully classified with 0.88 and 0.89 F-measure accuracies in 10-fold cross validation (10xCV) and leave-one-out (LOO) approaches, respectively. Sensitivity and specificity were 0.90 and 0.89 for LOO; 0.83 and 0.83 for 10xCV. Many of the 30 selected clusters are highly relevant to the addictive process, including regions relevant to cognitive control, default mode network related self-referential thought, behavioral inhibition, and contextual memories. Relative hyperactivity and hypoactivity of regional cerebral blood flow in brain regions in cocaine-dependent participants are presented with corresponding level of significance.

**Conclusions:**

The SVM-based approach successfully classified cocaine-dependent and healthy control participants using voxels selected with information theoretic-based and statistical methods from participants’ SPECT data. The regions found in this study align with brain regions reported in the literature. These findings support the future use of brain imaging and SVM-based classifier in the diagnosis of substance use disorders and furthering an understanding of their underlying pathology.

**Electronic supplementary material:**

The online version of this article (doi:10.1186/s12859-016-1218-z) contains supplementary material, which is available to authorized users.

## Background

Medical imaging techniques have dramatically improved our ability to explore the neural processes relevant to psychiatric disorders. These techniques can be group into two classes based on type of measurements: direct and indirect. Electroencephalography (EEG) and magnetoencephalography (MEG) are non-invasive modalities and directly measure electric changes associated with neural activity in the brain. A major limitation within these EEG and MEG are that they can only sense the electrical activity and magnetic fields oriented perpendicular to the surface of the brain and face the challenge of identifying the source of the underlying signal. While they have superb temporal resolution, their spatial resolution is limited.

Magnetic resonance imaging (MRI), functional magnetic resonance imaging (FMRI), positron emission tomography (PET), and single-photon emission computed tomography (SPECT) are the major approaches utilized in neuroimaging studies and indirectly measure neural activity. MRI/fMRI is the most widely used method in the brain imaging because of its low risk for subjects, better temporal and spatial resolution relative to other indirect neuroimaging methods. PET measures blood flow in the brain by injecting small amounts of radioactive tracer. Then, the accumulation of the tracer is scanned. Similar to PET, the modality of SPECT uses radioactive tracers and a gamma camera to construct two- or three-dimensional images with the computer support. SPECT scanners are more affordable that PET scanner. Both PET and SPECT can also be used to assess specific neurotransmitter receptor binding potential and functioning. Many studies have exploited these modalities in brain research and addiction [1].

However, these discoveries have not been either specific or sensitive enough to assist in the diagnosis or treatment of psychiatric disorders. Thus, the identification of persons either at risk of or suffering from most psychiatric disorders, including substance use, schizophrenic, affective, and anxiety disorders, remains dependent upon descriptive signs and symptoms. Brain imaging obtained from healthy and non-healthy groups can be analyzed via data-driven machine learning and data mining algorithms to elicit the key difference between subject groups. The findings may pave the path for identifying new neural mechanisms underlying these disorders as well as detecting those at risk or responsive to specific treatment approaches.

Support Vector Machines (SVMs) are relative new multivariate machine learning / pattern classification algorithms which have been intensively studied and benchmarked against a variety of techniques [2]. An SVM [3–5] classifier seeks maximum margin separation in multidimensional (multivariate) feature space in order to separate two classes with minimum error and has generalization power and feature mapping advantages over other classifiers such as Bayesian, Neural Networks, and Decision Trees. The paramount advantage of SVM classifiers over linear methods (e.g. discriminant analysis, perceptron, neural networks) is the use of a function to map original data to another multidimensional space in which linear separation yields more accuracy [3]. SVMs also offer a great deal of flexibility in that they can learn from multivariate subject data (continuous or categorical) such as demographic or clinical measures, gene expressions, or cognitive measures.

This *intelligent software* has been used to detect brain diseases, such as schizophrenia [6–8], Alzheimer’s disease (AD) [9–12], Huntington’s disease [13], attention deficit/hyperactivity disorder (ADHD) [14–16], Parkinson disease [17], and social anxiety disorder [18]. Classification accuracies of these studies vary between 55 and 100 % for two-category classification of healthy control vs diseased. One of most successful classification studies [10] used linear SVMs to classify patients with AD from four different groups (28 to 90 subjects per group) via T1-weighted anatomic MRI scans. In addition to the successful classification of AD and control participants, this technique was able to distinguish patients with mild AD from control subjects, and subjects with AD from those with frontotemporal lobar degeneration. The subjects were correctly assigned to the appropriate diagnostic category in 95 % of trials with 95 % sensitivity and 95 % specificity within LOO accuracy assessment method. As for substance use disorders, only alcohol-addicted subjects have been studied with similar data mining and machine learning algorithms [19–21] so far. Alcohol-dose effects on brain activation were explored using independent component analysis to isolate systematically non-overlapping networks and their time courses [22]. To our knowledge, there are no published studies presenting classification of cocaine dependence using SPECT data.

The primary impetus for the present study was to develop a clinically applicable framework to identify cocaine-dependent patients via brain imaging, using study participants assessed with single photon emission computerized tomography (SPECT) [23, 24]. The main aim of this study was to determine the brain regions to optimally classify cocaine dependents versus healthy controls using measures of regional cerebral blood flow (rCBF). We also wanted to explore whether the brain regions that classified cocaine-dependent vs. healthy controls would be related to cortico-striatal-limbic systems relevant to the addictive process [25–27]. On the other hand, the framework that was developed in this work does not depend on any particular experimental task, which means that the framework can be applied to and tested on SPECT data from studies which study other types of brain disorders.

## Materials and methods

### Participants and data acquisition

Ninety three two- to four-week abstinent cocaine-dependent and 69 healthy control participants, 24 to 48 years old, were studied (see Table 1). All participants underwent a medical history and physical examination, Structured Clinical Interview for Diagnostic and Statistical Manual of Mental Disorders-Fourth Edition (DSM-IV), clinical laboratory tests and urine drug screen. T1-weighted MRI scans were obtained from all but the first 20 subjects (10 cocaine-dependent) to enhance SPECT registration and rule out anatomic abnormalities. Financial compensation was provided to the participants for their involvement. Approval for the study was obtained from the Institutional Review Boards of the University of Texas Southwestern Medical Center at Dallas and the VA North Texas Health Care System.

**Table 1:** Demographics of participants

	Controls (*n* = 69)		Cocaine-dependent (*n* = 93)	
	Mean/n	SD/%	Mean/n	SD/%
Age (years)	34.6	7.5	40.0	6.8
Male	33	47.8 %	67	72 %
Race				
White	36	52.2 %	22	23.7 %
Black	20	29.0 %	69	74.2 %
Asian	3	4.3 %	0	0.0 %
Hispanic	10	14.5 %	2	2.2 %

Cocaine-dependent subjects were recruited from patients obtaining residential treatment for cocaine dependence at the VA North Texas Health Care System in Dallas, Homeward Bound, Inc. and the Nexus Recovery Center. All cocaine-dependent participants endorsed cocaine as their primary drug of choice. Cocaine-dependent participants were hospitalized as soon as possible after their last reported use of cocaine and remained in a structured, residential unit until the initial scan was completed. Participants were excluded from participation if they took any central nervous system active medications (including all psychotropics) or had any major medical or neurological disorders, active affective, anxiety or psychotic disorders (non-substance related), Axis I disorders, or organic brain syndrome. Women were all premenopausal. A negative pregnancy test was obtained on all female subjects prior to SPECT scanning.

Healthy controls were recruited through local ads in newspapers, the internet and notices on bulletin boards. Exclusion criteria for healthy controls included the criteria as noted for the cocaine-dependent subjects, as well as a lifetime history of substance use or other Axis I disorder (except nicotine dependence). Healthy controls with a first-degree relative or two or more second-degree relatives with a substance-use disorder were also excluded.

Study sessions took place in the afternoon at the Nuclear Medicine Center or the Clinical Trials Office at the University of Texas Southwestern Medical Center at Dallas. Participants from three studies were included: Study I) Subjects (37 controls, 35 cocaine-dependent) participated in two study sessions to assess limbic sensitivity to the local anesthetic procaine [28, 29]. Saline was administered in the first session. Subjects were blinded to condition. Study II) Subjects (20 controls, 25 cocaine-dependent) participated in four sessions to assess cholinergic and 5HT3 receptor systems. Saline was administered in one of the four sessions; study order was double-blind and randomized [30, 31]. Study III) Subjects (12 controls, 33 cocaine-dependent) were assessed at rest [32].

SPECT images were acquired on a PRISM 3000S three-headed SPECT camera (Picker International, Cleveland, OH, USA) using low energy ultra high-resolution fan-beam collimators (reconstructed resolution of 6.5 mm) in a 128 × 128 matrix in three-degree increments. For each scan, 20 mCi of ^99m^Tc HMPAO was administered, and total scan duration was 23 min. Image reconstruction was performed in the transverse domain using back-projection with a ramp filter. The voxel size in the reconstructed images were 1.9 mm^3^. Reconstructed images were smoothed with a fourth-order Butterworth 3-D filter, attenuation corrected using a Chang first-order method with ellipse size adjusted for each slice.

To register SPECT images more accurately, a rigid-body co-registration of the SPECT scan to a skull-stripped T1-weighted high-resolution (0.8 × 0.8 × 1.5 mm) structural MRI scan of the same subject transformed the SPECT image into the same space as the MRI. Spatial transformation parameters were then calculated using the statistical parametric mapping (SPM5) to warp the MRI into standard MNI [33] space. The same transformation was then applied to the co-registered SPECT image and output images were resliced to 2 mm^3^ voxels. All images were smoothed to a final resolution of 10 mm and the voxel signal values normalized to whole brain counts (to correct for individual variability in global cerebral blood flow). All scans were combined and mapped into 2D matrix where each column was a subject and each row was a feature (voxel). In this representation all non-mask voxels were eliminated, reducing the feature space twofold. All statistical analyses were carried on a 64-bit 3.0 GHz PC using MATLAB scientific programming language [34]. In the reporting of brain regions, we used Automated Anatomical Labeling [35] (AAL, 90 regions, only cerebrum) atlas with the dimension of 79 × 95 × 69 voxels.

### The framework

We designed the framework in which the input is the normalized SPECT data of participants from both groups, cocaine-dependent and healthy control. The framework here is not task-dependent, which means that the classification framework is also applicable to other similar neuroimaging studies. Since all SPECT images were normalized to AAL mask, it is proper to consider a voxel (intensity of rCBF changes) as a feature at the very low level, representing the subject. Therefore, an array of voxels from the same spatial location in 3D for each participant represents one-dimension of the multidimensional classification space. Through this study, the term of *feature* is used for the voxels in the cerebrum.

**Fig. 1:**
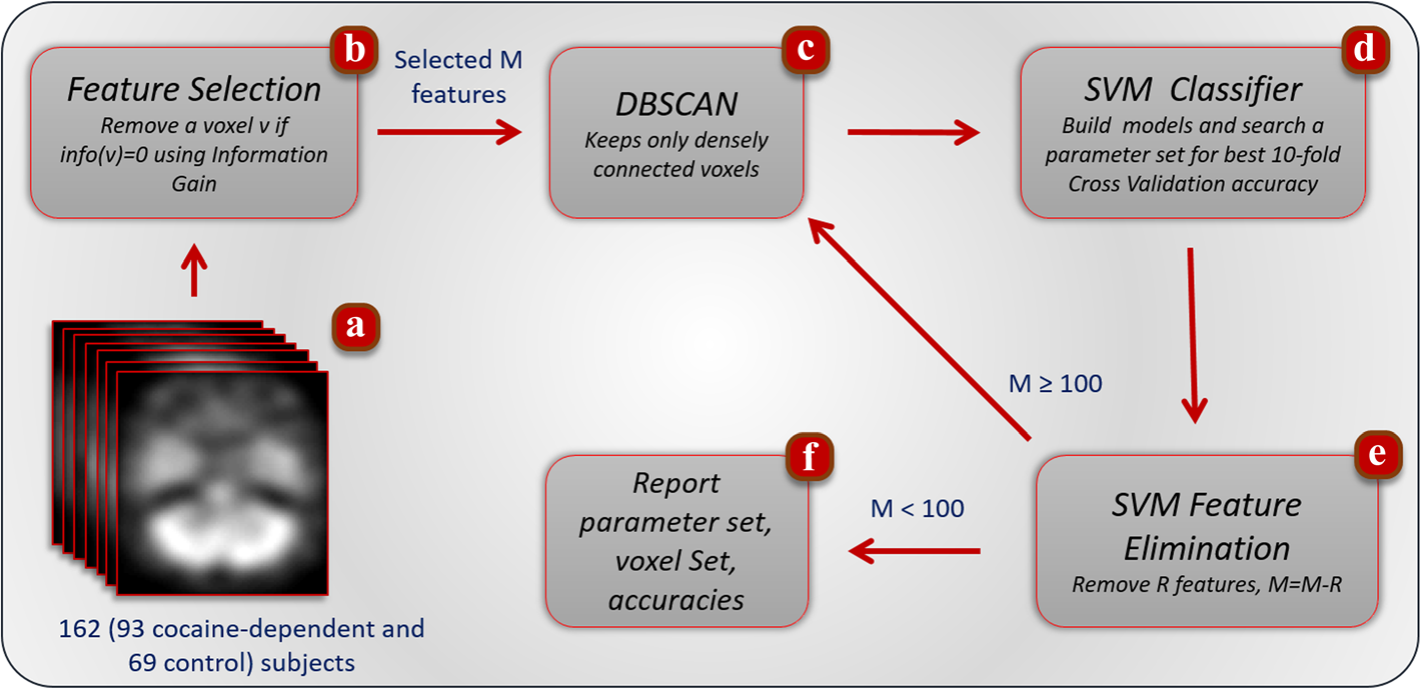
Feature selection, parameter selection, classification, and reproducibility framework. To find the best classification model, the framework is started with single photon emission computerized tomography (SPECT) scans (162 subjects) (**a**). Information Gain algorithm (**b**) removes non-informative voxels. A loop of parameter selection and Support Vector Machines (SVMs)-based feature selection then takes place. Only voxel clusters with size ≥20 are kept in the dataset (**c**) with DBSCAN, a density-based clustering method. At each iteration, the dataset is trained and tested (**d**); SVM’s feature elimination (**e**) refines voxels before next DBSCAN run. When there was no more than 100 voxels, parameter search was ended. At the next steps, model, 10-fold Cross Validation (10xCV), and leave-one-out (LOO) classifications were carried out and accuracies and set of selected voxels were identified (**f**)

Referring to Fig. 1, we first eliminated all non-AAL mask voxels from the data set of 162 participants (Fig. 1a). After the elimination, the imaging data set was represented as a 2D matrix, where one dimension is used for voxels (features), and the other dimension denotes the participants (samples). Numerous non-informative voxels were eliminated using Information Gain method (Fig. 1b) in order to reduce the feature space. After this step, individual voxels which are not a member of a connected cloud of voxels (groups) were iteratively removed (Fig. 1c). The classification accuracy was assessed with an SVM classifier (Fig. 1d); and the least significant *R* voxels (Fig. 1e) were removed, where *R* was set to 100 empirically. The loop of sub-sections Fig. 1c–e continues to run until there is no voxel to be used in classification (Fig. 1d). Further details of the framework are described in the following subsections.

We considered Principal Component Analysis (PCA) as a feature selection method before the other information-thoretic approach. However, the issue of computational complexity of PCA made us search for another method. Note that the voxel size of mask is *N* = 203,632, and we had *M* = 162 participants. So, the covariance matrix *C* ∈ ℝ^*NxN*^, which has time complexity of O(MN^2^). Furthermore and addition computation with O(M^3^) required for singular value decomposition applying to resulting matrix of first step. A PCA is also requires a great amount of the memory for the matrix calculations. We did experiments to find principle component and received this error: *Matrix of 203633 x 203633 = 41466398689 elements is too large to be allocated using a single Java array*. Note that 203633 = 203632 + class variables with 4-byte unit size requires around 155 GB memory to calculate covariance matrix and following eigenvalue calculations, which is impractical with a mediocre computer.

### Information gain

The whole unprocessed dataset consisted of a concatenated 162 × 203,632 matrix from all of the brain scans. A SPECT image of participant in the dimension of 79 × 95 × 69 = 517,845 voxels, which leaves 203,632 voxels after brain extraction and thresholding using the AAL brain atlas. This meant that we should device a classifier to deal with all 203,632 features at-large, which is practically infeasible. As done in many classification frameworks [6, 9–12], we reduced the number of voxels by selecting only the most informative ones. To find out the optimal analysis for initial voxel selection, the Gaussian distribution of each voxel over all sample was investigated first. Since we found out that only 12.6 % of 203,632 voxels were normally distributed with the method proposed by Lilliefors et al. [36] in both groups, we opted not to use the traditional statistical methods to reduce the size of features. *Information Gain* [37], an information theoretic-based feature reduction algorithm, was employed in this step. As a result, 6,683 of the 203,632 brain voxels were identified as significantly informative in the classification of the two groups of subjects. Information Gain, also known as Kullback–Leibler divergence, is a non-parametric method used to select a feature that reflects minimum randomness in class distributions. More formally for a two-class problem, it is given as

*IG*(*v*) = − *p*_1_ log_2_ *p*_1_ − *p*_2_ log_2_ *p*_2_,

where, *p*_1_ and *p*_2_ are the probabilities that the voxel *v* belongs to class 1 and 2, respectively. This first step of entropy-based voxel selection served as a blind dimension selection and discarded all but voxels with *IG*(*v*) > 0 regardless of the spatial or informational correlation between pairs of voxels (Fig. 1b).

### Clustering voxels in 3D

Following the removal of many features with Information Gain, we are left with 6,683 voxels that are from different locations from the AAL bring regions. Before the classification step, we removed individual voxels which are spatial proximity of a group of other selected voxels. We wanted to determine the minimum cluster size (number of spatially connected voxels) which would provide an overall false discovery rate of 0.01 and a voxel-level false discovery rate of 0.002. We used the AlphaSim utility of AFNI software which runs Monte-Carlo simulations, and we determined cluster size to be 20 [38, 39].

**Fig. 2:**
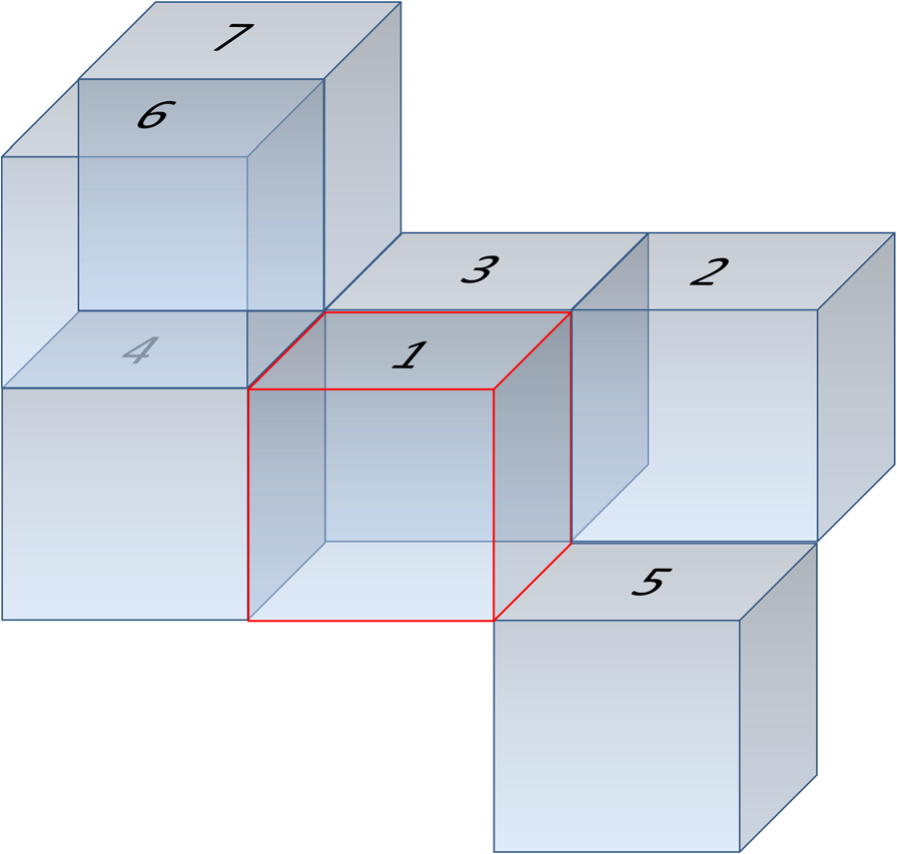
During the expansion of clusters (clouds of spatially connected voxels) in 3D, a cluster can grow via those voxels that are sharing a common edge with the one of existing cluster’s voxels. This property is regulated by parameters of $\epsilon =\sqrt{2},\  Minpts=2$ . In this 3D figure, each cube represents a voxel. The centered (red-outlined) voxel (#1) and only five (#2, #3, #4, #5, #6) of its twelve common-edge neighbors are depicted for the sake of simplicity. Note that point-based neighborhood (sharing only one corner, such as #1 and #7) between two voxels does not satisfy the condition of cluster expansion

A spatial case of *A density-based algorithm for discovering clusters in large spatial databases with noise* (DBSCAN) [40] with $\epsilon =\sqrt{2},\kern0.75em  Minpts=2$ was used. The *ϵ* and *Minpts* are two parameters for DBSCAN clustering algorithm to fine-tune how far a boundary of a cluster can go and how dense at least each cluster can be, respectively. The conditions of $\epsilon =\sqrt{2},\kern0.5em  Minpts=2$ requires that the minimum size cluster be two and these two voxels should be next to each other sharing a common edge $\left(\epsilon =\sqrt{2}\right)$ in 3D space (see Fig. 2).

**Fig. 3:**
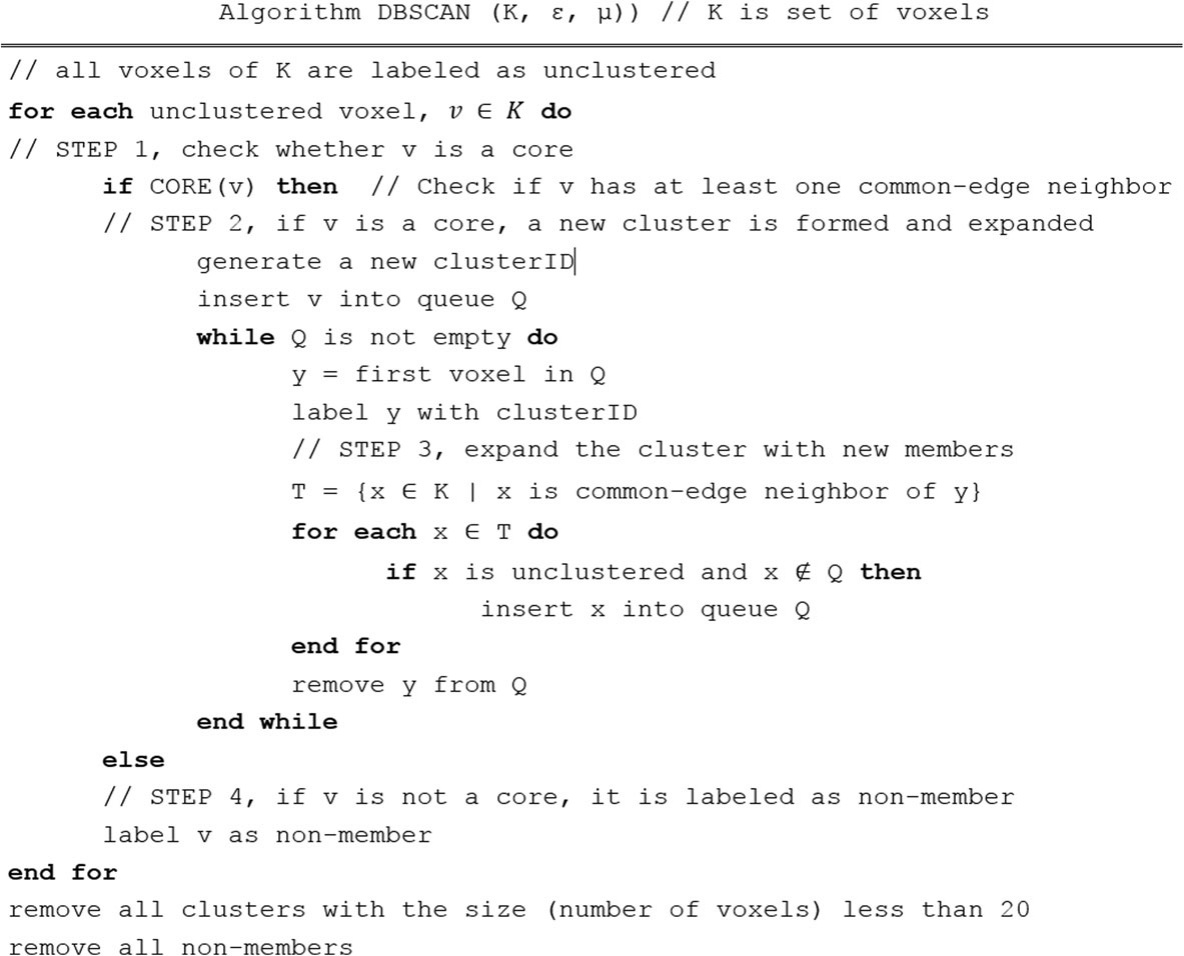
The pseudocode of the modified DBSCAN algorithm to find group of voxels through the processes of feature selection

Let *K* be set of voxels resulted from either first feature selection, Fig. 1b or SVM feature elimination step, Fig. 1e. The pseudocode of a special case of DBSCAN is presented in Fig. 3. It performs only one pass in the set of voxels, *K*, and finds all clusters under a given parameter conditions above. At the beginning all voxels are labelled as *unclustered*. For each voxel that is not yet clustered, DBSCAN checks whether this voxel, *v*, is a core (Step 1). This is simply to check if *v* has at least one common-edge neighbor. If the voxel is a core, a new cluster is expanded starting with this voxel (Step 2). Otherwise, the voxel is labelled as a non-member (Step 4). To expand an existing cluster, DBSCAN begins by inserting all common-edge neighbors of the initial voxels into a queue (Step 3). For each voxel, *y*, in the queue, the algorithm finds all common-edge neighbors of *y* and inserts only voxels that are unclustered yet and not the member of queue into the queue. This is repeated until the queue is empty. Since each voxel of a cluster is labelled with a *clusterID*, they are not process again in the later stage of the algorithm. The DBSCAN algorithm labels each voxel either a member of a cluster or a non-member. At the end, all non-members voxels and members of a cluster with less than 20 voxels are removed from the data set. For instance, in the first run of DBSCAN algorithm, 1164 out of 6,683 voxels are removed data set either because of they are not a member of any cluster or they could not form big enough clusters (<20).

### A statistical classifier: support vector machine

From the machine learning perspective, classification is the process of mapping a new data sample (a participant) to one of known labels where rules or functions are induced from a training population. In this study, the size of training data was 162 – *round*(162 × 0.1) = 146 participants in the case of 10xCV, and 162 – 1 = 161 in LOO. Training and test datasets were normalized between −1 and 1. Identification of cocaine-dependent participants in a cohort with healthy controls is a binary classification and it was carried out via the SVM statistical classification algorithm, SVM. Note that in this study SVM was used as both classification tool and feature selection method. In the classification phase (Fig. 1d), all voxels from the dense cluster found with DBSCAN clustering algorithm were fed to SVM as a feature set.

**Fig. 4:**
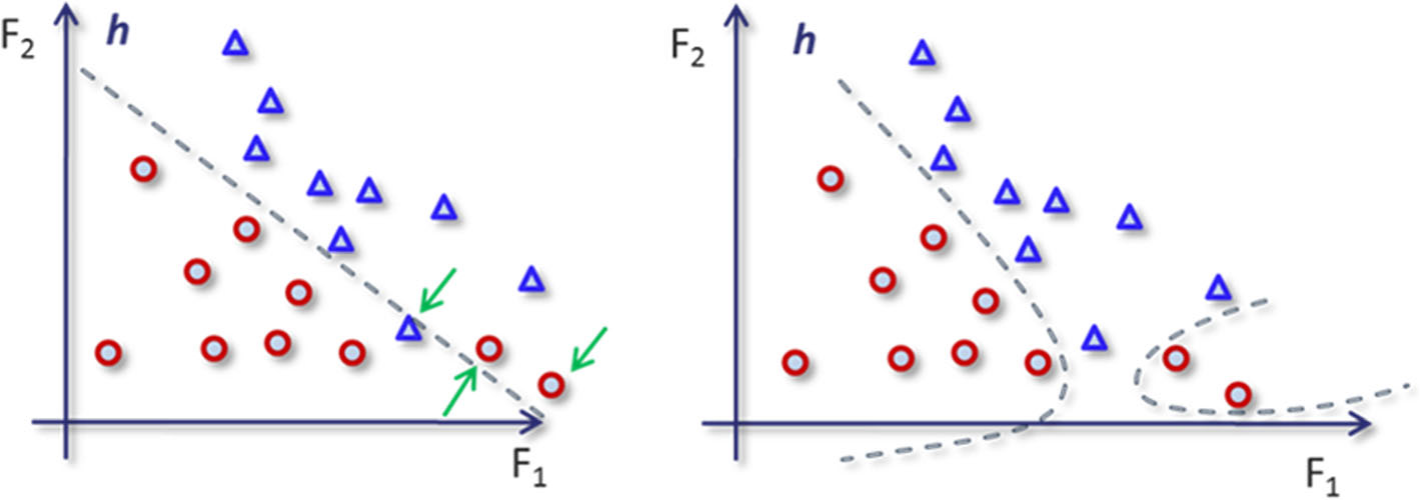
In this toy illustration, the hyperlines (dashed lines) of Support Vector Machine (SVM) separate cocaine-addicted (red circles) from healthy control (blue triangles) participants via two features F_1_ and F_2_. Left panel: The kernel function, which maps a data point to another dimension, is in the form of Φ(x). Φ(x^'^), which produces a linear decision boundary. Right panel: The separating line is non-linear, since a polynomial kernel, ((Φ(x). Φ(x^'^))^4^, is used to map the data. In this case, the decision boundary is non-linear, placing more cocaine-addicted participants in the correct regions. For instance, two of the cocaine-addicted participants and one healthy participant pointed with green arrows are misclassified with a linear kernel. Once trained with a polynomial kernel, the decision boundary is more flexible resulting in fewer misclassified participants. The use of the polynomial kernel increases the accuracy

**Fig. 5:**
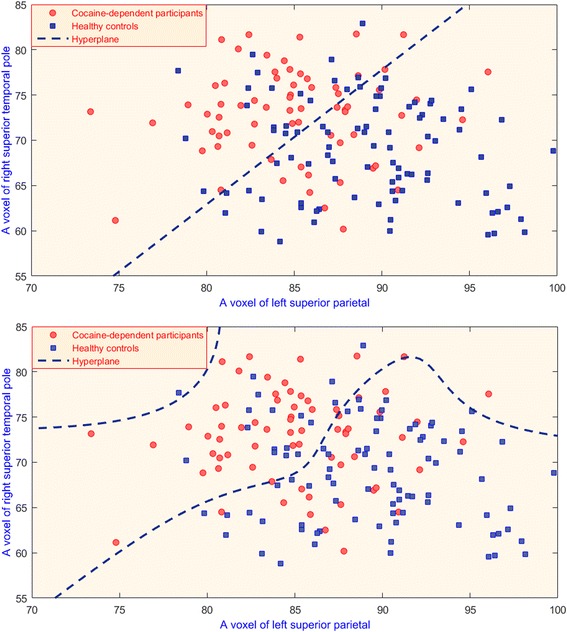
The hyperplanes (dashed line) of SVM model separating patient groups via two voxels from right superior temporal pole (vertical axes) and left superior parietal (horizontal axes) with different kernel functions. (upper) The kernel function was in the form of **Φ**(**x**). **Φ**(**x** ^'^), which produces a linear decision boundary having 117 out of 162 subjects were correctly classified. (lower) The separating line is non-linear since a polynomial kernel ((**Φ**(**x**). **Φ**(**x** ^'^))^4^ was used to map the data. 120 out of 162 subjects were correctly classified

In many SVM classification problems the resulting classifier cannot be visualized because of high dimensionality. For instance, given the fact that we worked on this study with thousands of voxels, it would be impossible to present the classifier for human perception. Hence, a toy example with only two features and 19 samples (10 circles and 9 triangles which constitute the two categories/groups) are depicted in Fig. 4, where the resulting hyperlines of linear and polynomial kernels of SVMs are shown to visualize kernel effect in classification. Furthermore, two SVM models classifying all of the participants with only two features (voxels) are visualized in Fig. 5. In each sub-figure, the resulting separating line (hyperplane) with corresponding training kernel is shown. The accuracy of obtained models were not same. In Fig. 5-upper, the problem space was divided into two sub-regions and yielded an accuracy of 0.72 (number of correctly classified participants is 117). However, in Fig. 5-lower, hyperplane is polynomial to include more patients in correct regions. For instance, the subject with the left superior parietal (horizontal axes) expression around 96 and right superior temporal pole (vertical axes) around 78 was misclassified with linear kernel. However, the same subject was correctly labeled as cocaine-dependent once SVM classifier was trained with a polynomial kernel. Particular to this comparison, the mapping of data with a polynomial kernel increased the model accuracy of the system from 0.72 to 0.74, meaning that three more participates are correctly classified. An SVM classifier labels the group/category membership (in our case, as either cocaine-dependent or healthy) by defining a hyperplane in multi-dimensional space, separating group-specific features (see Fig. 4). However, the large number of voxels in SPECT images in our case creates unmanageably high dimensionality, requiring that only a subset of selected features is used in the classification algorithm. Therefore, the framework introduced in Section 2.2 includes two levels of feature selection schema: 1) dimension reduction with Information Gain reduced the number of voxels to a manageable set, i.e., from 203,632 to approximately 5,500 voxels (see Fig. 1); 2) SVM-based feature selection reduced size of voxels from 1000s to the order of 100 s (see Fig. 1e), iteratively.

Through these iteratively refined steps, the most significant voxels remained in the dataset by removing the less significant ones. At each step with the refined voxel set, a new SVM classifier was trained to separate controls from the cocaine-dependent participants. Because of the nature of SVM’s heavy dependence on parameter selection, in each iteration, training and classification were done using various parameters and kernels. The list of kernels and parameters are given in Table 2. The classification was performed using all of the parameter combinations listed in Table 2, and the parameter combinations which yielded the best classification accuracy result are reported in the Results section below.

**Table 2:** List of various parameters used with SVM on the dataset

Kernels	Linear, radial basis function, polynomial, sigmoid
SVM type	C − SVM, ν − SVM
Degree of polynomial function	3, 4, 5, 6, 7
*C*	2, 4, 10, 12, 15, 20 (N/A in ν − SVM)
Gamma (*γ*)	0.001, 0.003, 0.01, 0.03, 0.05, 0.1
Coefficient	0.01, 0.1, 1, 5, 10, 15, 20
Nu (ν)	0.2, 0.29, 0.4, 0.5

Since one of the aims is to elicit brain regions that significantly contribute to the SVMs classifier, we kept all 162 subjects in the main loop of the framework. Once we determined the best-possible parameter and voxel sets, their classification power was evaluated with LOO and 10xCV methods.

### Support vector machine based feature selection

In a classification framework, features (e.g., the selected voxels in this study) are information carrying representatives of samples (e.g., study participants). In this context, feature selection involves removal of insignificant features aiming for a better classification accuracy with the remaining features. For the feature selection sub-section in the loop of framework, an SVM-based approach was adapted since SVM is being used in the classification of controls and cocaine-dependent participants in the previous step. Guyon et al. [41], for example, showed that their recursive feature elimination technique utilizing SVM yielded better accuracy than correlation-based methods in a DNA microarray dataset, which is similar to this study in terms of high dimensionality of voxel data set and the machine learning task of classification of control and diseased subjects.

The feature elimination framework started with the set of possible significant features to be used in the classification algorithm (Fig. 1d). This set of features was then refined through the elimination of non-significant ones from the initial set. Features to be removed were selected utilizing weight vector ***w*** ∈ ℝ^***n***^ of linear SVMs. Since each component of ***w*** corresponds to a feature in the classification problem, Guyon et al. [41] showed that the larger |***w***_*j*_|, *j* ≤ *n* , the more contribution to decision in the classification. In the previous notation, *n* is number of voxels and |***w***_*j*_| is the length of *j* ^th^ component of the weight vector ***w***. After each classification attempt in the framework, a linear SVM classifier was run and 100 least significant features were removed from the data set. When comparing to the Information Gain feature selection method which was used in the first step to remove vast number of voxels, the one proposed by Guyon et al. is more refined, i.e. more successful in sorting out the voxels which contribute most to the classification.

### Classification software and accuracy

LibSVM [42] was used for the training of SVM models and classifications of participants. The accuracies obtained with 10xCV and LOO assessment methods along with sensitivity and specificity are reported. In 10xCV, the dataset (*N* = 162 participants) was divided into 10 non-overlapping quasi-equal class distribution partitions. In each of the 10 folds, one partition (16 participants) was held as test data, *S*, while a model was built with the remaining nine partitions (training samples, *N – S*). *S* is also called the validation set. Through this method, every participant is entered in the test set one time, and in the training set nine times. Finally, the average of accuracies from each fold was reported.

The LOO is the exhaustive version of *k*-fold cross-validation with *k* = *N* = 162, and it simply avoids combination-driven calculation problem of *k*-fold cross-validation. In LOO, we exclude only one test subject, *S* = 1, from the group of *N* = 162 and classify whether *S* is dependent or healthy using the model built based on the remaining *N – S* = 161 subjects, which constitute the training group. In turn, each subject is considered as *S* once, and this classification process is repeated *N* = 162 times for each of the subjects. The accuracy of model is reported in each case (0 or 1 in this case), and an average of all 162 accuracies is reported. Note that SVM classification models obtained for each training dataset result in different but similar classification model (a hyperline in multidimensional space) even if the SVM is trained with exactly same parameters and constraints. In general, a dataset with significant informative features would be more robust to removal of a particular *S* assuming that the other subjects who are of the same class as *S* will cover the missing information excluded by the removal of *S*.

F-measure was the criterion to choose the best classification model. F-measure, extensively used in information retrieval domain, is the harmonic mean of *precision* and *recall*. Recall is the percentage of positive labeled instances that were predicted as positive and found by *True Positive*/(*True Positive* + *False Negative*). Precision is defined as the percentage of positive predictions (e.g., cocaine-dependent) that are correct, and calculated as *True Positive*/(*True Positive* + *False Positive*). Based on given ratios, the F-measure was calculated as $$ \frac{2*\mathrm{Precision}*\mathrm{Recall}}{\mathrm{Precision}+\mathrm{Recall}} $$. If the same F-measure is obtained from several SVMs, the one with highest recall is selected.

## Results

### Feature selection and classification accuracy

**Fig. 6:**
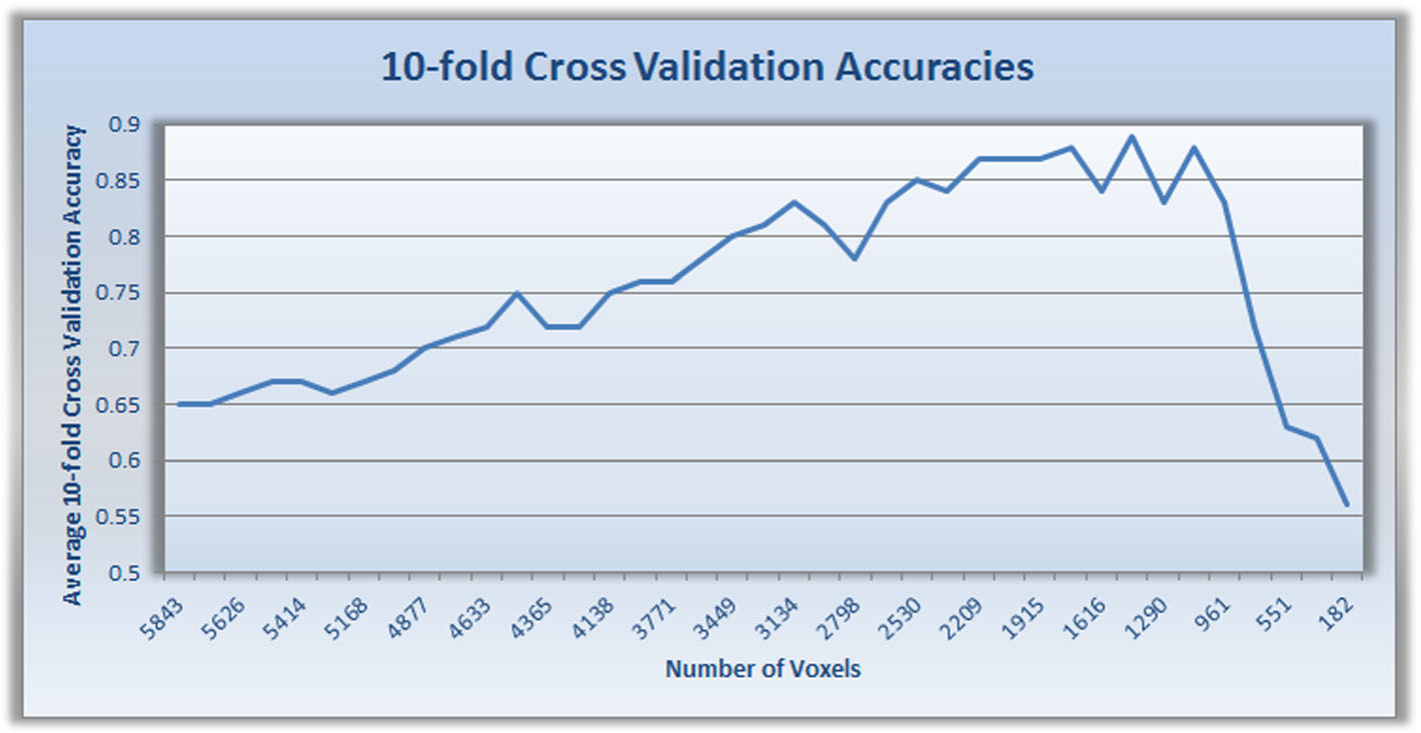
10-fold cross-validation accuracies (the accuracy values used to select best parameter and voxel sets). These result are obtained with Support Vector Machines (SVM), polynomial kernel, *f*(*x*) = 0.007(Φ(x). Φ(x^'^) + 10)^4^, coefficient *R* = 1.0, and penalty parameter *C* = 15. The 10-fold cross validation accuracy peaks with 1500 voxels in 30 clusters

Since only 12.6 % of the 203,632 voxels were normally distributed, Information Gain returned only 6,683 voxels as a starting set (Fig. 2b). While the voxels are iteratively refined in the loop of the framework, it was found that polynomial kernel, *f*(*x*) = 0.007(Φ(x). Φ(x^'^) + 10)^4^ with coefficient *R* = 1.0 and penalty parameter *C* = 15 within a *C* ‐ SVMs yielded the best average classification (F-measure) accuracy using 1500 voxels. Figure 6 shows how average accuracy changed over number of selected voxels.

**Fig. 7:**
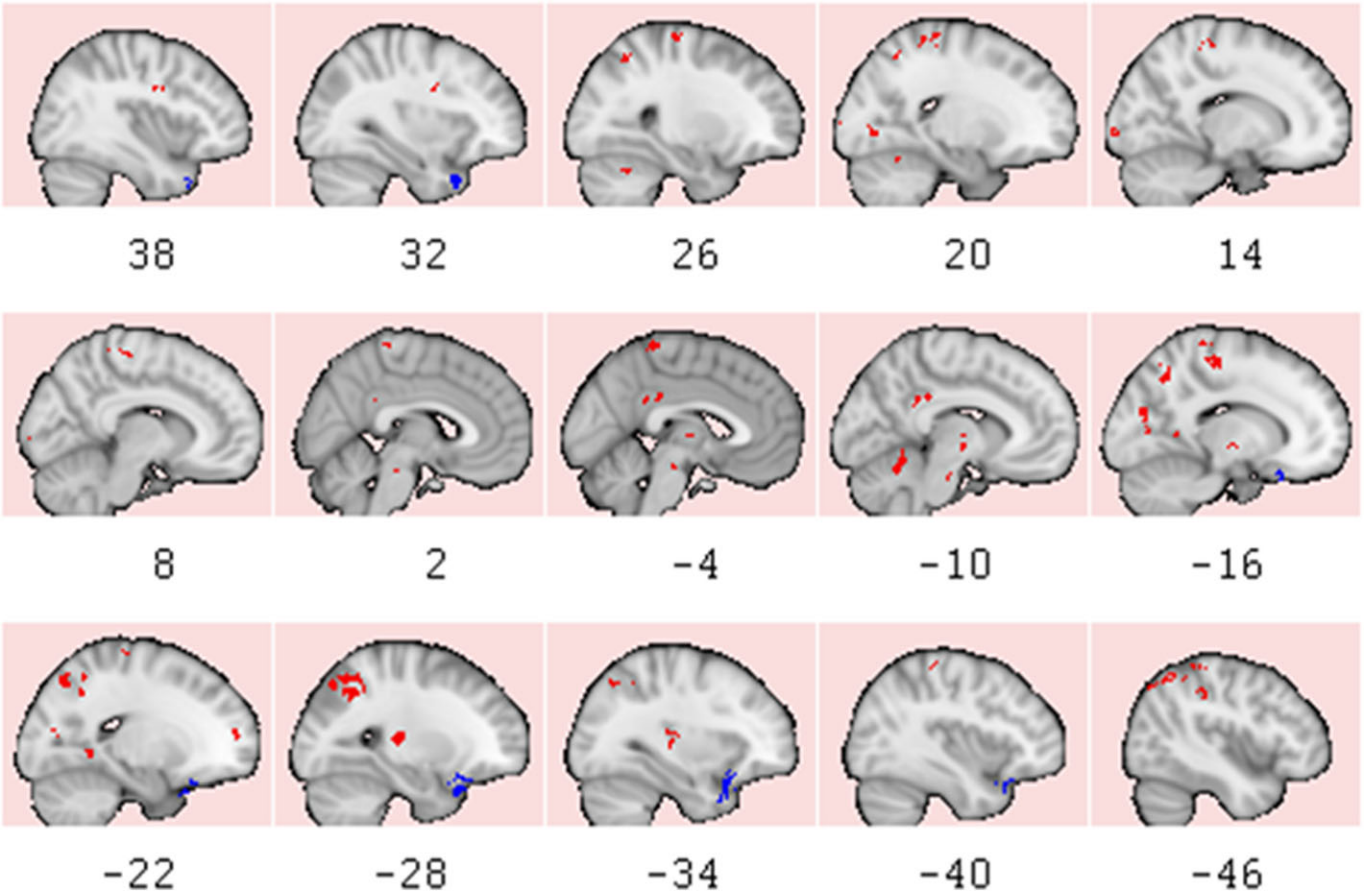
Regions that is used to classify cocaine-dependent and controls participants with the best accuracy. 1500 voxels in 30 clusters were identified. Figure shows sagittal sections of region-of-interests (ROIs) where 100 % model, 89 % LOO, and 88 % 10xCV accuracies were obtained. Red identifies clusters of increased regional cerebral blood flow (rCBF) in cocaine-dependent participants relative to controls. Blue identifies clusters of decreased (rCBF) in cocaine-dependent participants relative to controls. Slice numbers are in MNI coordinates. MNI coordinates of each cluster and images in axial planes are provided in the Additional file 1

To select the parameter set for SVM and voxel clusters, three different types of schemas, focusing 1500 voxels in 30 clusters with at least 20 spatially connected voxels in each, were explored. Model accuracy for all assessments (F-measure, sensitivity, specificity) was 1.0, meaning that both groups were perfectly separable in a higher dimensional space, which had all 162 participants mapped by a degree-four polynomial kernel. The F-measure of LOO and 10xCV were 0.89 and 0.88, respectively. Sensitivity and specificity were 0.90 and 0.89 for LOO; 0.83 and 0.83 for 10xCV, respectively. Similar results for LOO and 10xCV indicated that the classification model build using 1500 clustered voxels appeared robust to the exclusion of either one or 10 subjects. 29 of 30 clusters showed significant features having *p*-value less than or equal to 0.002. All identified clusters and corresponding regions are detailed in the Fig. 7.

### Identified regions of interest

Of the 30 clusters used to successfully classify cocaine-dependent and control participants, 27 showed relative rCBF increases in cocaine-dependent compared to control participants and three showed rCBF decreases in cocaine-dependent compared to control participants (see Fig. 7; transverse images and MNI coordinates provided in Additional file 1). A large cluster in the left superior parietal gyrus, encompassing almost 20 % of the voxels used in classification, showed higher rCBF in the cocaine-dependent participants relative to controls. Other clusters of increased rCBF in the cocaine-dependent participants included the right and left pre- and post-central gyrus and cerebellum, the left transverse temporal gyrus, inferior parietal lobule, thalamus, parahippocampus, posterior cingulate, and cuneus, and right middle temporal gyrus, lingual gyrus and precuneus. Clusters with decreased rCBF in the cocaine-dependent participants, relative to controls, were restricted to the left lateral OFC and bilateral superior temporal cortex.

## Discussion

It is shown that a machine learning framework based on SVM-based classifier and feature selection method and primarily supported with a density-based clustering tool successfully classified cocaine-dependent from healthy controls individuals with 0.89 LOO and 0.88 10xCV accuracies. Sensitivity, the ability to correctly identify those having the disorder, was 0.90. Given these high classification rates, determined by cross-validation, our final SVM model may offer insights into the pathogenesis of cocaine addiction.

Several clusters successfully classifying cocaine-dependent participants and healthy controls are highly relevant to the addictive process, including regions relevant to cognitive control (e.g., superior parietal cortex) [43], default mode network related self-referential thought (e.g., posterior cingulate cortex, precuneus) [44], behavioral inhibition (e.g., lateral OFC) [45], and contextual memories (e.g., parahippocampal gyrus) [46]. Perhaps of equal note are some regions intimately associated with the addictive process that were not identified in the classification process (e.g., striatum, ventromedial OFC, dorsolateral prefrontal cortex, anterior cingulate cortex, and amygdala). Similarly, hyperactivity of prefrontal cortex in addiction subjects was reported in [47, 48]. In our attempt to limit false positives, at least 20 spatially connected voxels were required during feature selection. Thus, smaller–but physiologically relevant–clusters may have been missed. Conversely, a number of clusters important to our classification did not encompass regions typically associated with addictive processes, highlighting the potential importance of a theoretical statistical approaches for identifying relevant–but unexpected–brain regions. Our findings, therefore, highlight the importance of utilizing whole brain analyses to identify regions useful in discriminating persons with addictive disorders from healthy controls. SVM classification of resting state functional connectivity has also been used to successfully classify heroin-dependent subjects and healthy controls, although the study population was limited to 25 participants [49].

Although classification was conducted in a binary fashion, i.e. positive (cocaine-dependent) or negative (healthy control), brain alterations may occur over the course of an addiction and may differ depending upon disease severity. Thus, an extension of the SVM approach could consider probabilistic classifiers in the future, allowing the identification of specific subgroups of addicted patients (e.g., those at high of low risk of relapse or those at variable intensity of addiction). Pariyadath et al., for example, has recently identified resting state neural networks predictive of nicotine dependence using an SVM-based classification approach [50].

Strengths of our approach included a relatively large sample of cocaine-dependent participants at least 2–4 weeks abstinent, precluding the acute and withdrawal effects of cocaine that confound imaging studies conducted during the first several days of abstinence. Participants were without other active DSM-IV substance dependent (except nicotine dependence) or psychiatric disorders and were not taking psychotropic medications. The spatial resolution (6 mm reconstructed resolution Niall planes) of the SPECT, 4 × 4 × 4 cubic mm voxel size, and 20 voxel cluster restriction provided a minimum cluster size well within the resolution of our device. Potential limitations included the use of highly selective populations dissimilar from typical clinical populations that may limit the generalizability of our findings. Also, the use of both saline and resting scans offers a possible confound, although we have previously reported similarities in rCBF during both scan in Cohort I participants [23]. Another limitation is that the features selection algorithms, both of information gain and SVM-based feature selection, have not been tested on a completely new sample of test subjects (that is, subjects that were part of the feature selection training set). Note that this problem only regards feature selection steps, not the classification algorithm. The LOO and 10xCV approaches remedy this problem and classification algorithm run on an entirely different test subject set. The two groups differed in gender, age, and race, although consideration of these potential confounds may minimally affect the model. Since other demographic variables were not consistently obtained over the span of time used to collect the three cohorts, other potentially relevant confounds (e.g., other substance use, socioeconomic status, education) were not available for inclusion.

Our findings support the use of machine learning statistical approaches in the classification of patients with substance use disorders. Coupled with structural and functional neuroimaging, this approach offers a powerful technique for distinguishing neural signatures of relapse, classifying features overlapping with and/or dissimilar from other psychiatric disorders, and potentially identifying neuroplastic alterations underlying these disorders.

## Conclusion

In this study it is presented that a generalizable machine learning framework can successfully classify cocaine dependent subjects using SPECT images. The brain regions associated with the best classification accuracy mainly point to some of the addiction related brain regions. In the future, disease state of cocaine dependency can be determined with a similar framework since the distance of each subject from a subject to hyperline, which is boundary to separate controls and dependent participates in multidimensional space, implies probability of being positive or negative in the classification. In a screening study, detecting those who are at risk or moving toward to decision boundary could benefit individually before they are acutely dependent.

This study was conducted with SVM classification and SVM-based feature selection algorithms. Although SVM is known as one of the most successful classifier for multidimensional dataset, in the future a methodological comparison study involving other classifiers (Random Forest, Decision tree, neural networks, Bayesian) and feature selection algorithms will be conducted.

## Additional file

Additional file 1: Transverse images and MNI coordinates for identified brain regions. (DOCX 336 kb)

## Abbreviations

10xCV: 10-fold cross validation
AD: Alzheimer’s disease
ADHD: Attention deficit/hyperactivity disorder
DBSCAN: Density-based algorithm for discovering clusters in databases with noise
DSM: Diagnostic and statistical manual of mental disorders
EEG: Electroencephalography
LOO: Leave-one-out
MEG: Magnetoencephalography
MNI: Montreal Neurological Institute
MR: Magnetic resonance
MRI: Magnetic resonance imaging
OFC: Orbitofrontal cortex
PCA: Principal component analysis
PET: Positron emission tomography
rCBF: Regional cerebral blood flow
ROC: Receiving operating characteristic
SPECT: Single photon emission computerized tomography
SPM: Statistical parametric mapping
SVMs: Support vectors machines
VA: Veteran administration
