# Supplementary material for: Successful classification of cocaine dependence using brain imaging: a generalizable machine learning approach
Source: BMC Bioinformatics. 2016 Oct 6;17(Suppl 13):357. doi: 10.1186/s12859-016-1218-z (PMC5073995; doi:10.1186/s12859-016-1218-z)
Supplement: Additional file 1: — Transverse images and MNI coordinates for identified brain regions. (DOCX 336 kb) [file 12859_2016_1218_MOESM1_ESM.docx]

**Additional file 1**

**
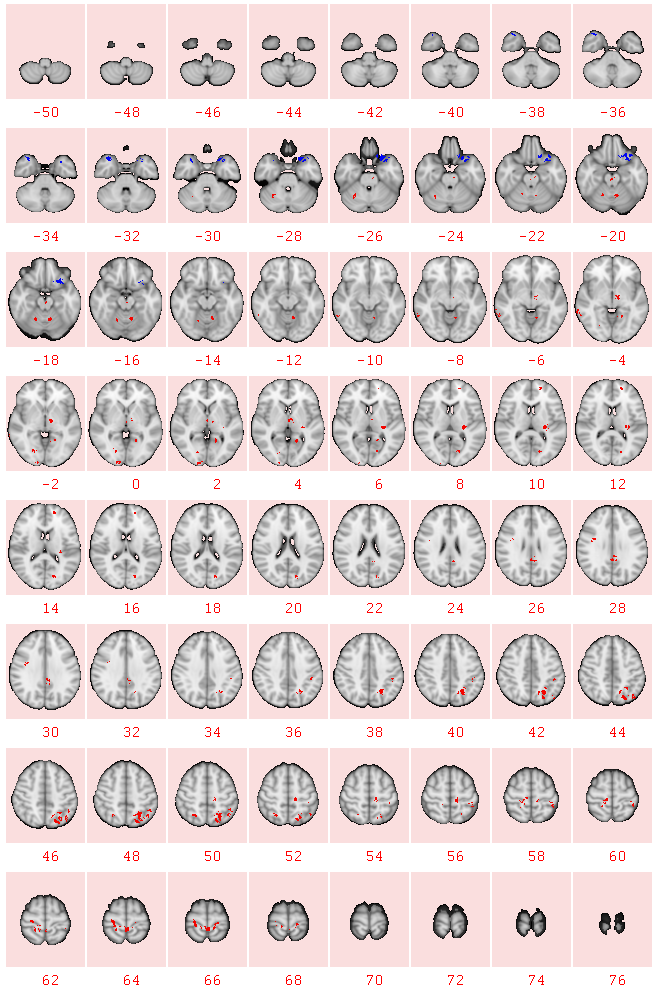
Figure S1.** 1500 voxels in 30 clusters in axial sections (R-L), which is used to classify cocaine-dependent and controls participants. Figure shows sagittal sections of region-of-interest within 1500 voxels, where 100% model, 89% Leave-one-out, and 88% 10-fold cross validation accuracies are obtained. Numbers are z-axis coordinates in MNI space. Red identifies clusters of increased regional cerebral blood flow (rCBF) in cocaine-dependent participants relative to controls. Blue identifies clusters of decreased rCBF in cocaine-dependent participants relative to controls. MNI coordinates are provided below.

| **Table S1.** MNI Coordinates of Clusters Identified in Figure S1. P-values for each cluster were less than 0.002. | | | | | |
| --- | --- | --- | --- | --- | --- |
| Cluster | | # of Voxels | MNI | | |
| **Increased rCBF** | |  | X | Y | Z |
| 1. | L superior parietal gyrus | 292 | -24 | -50 | 50 |
| 2. | L transverse temporal gyrus (Heschl’s gyri) | 68 | -32 | -32 | 10 |
| 3. | L cerebellum, posterior lobe | 66 | -10 | -56 | -14 |
| 4. | R postcentral gyrus | 65 | 22 | -30 | 68 |
| 5. | L cuneus | 55 | -20 | -72 | 8 |
| 6. | L inferior parietal lobule (angular gyrus) | 48 | -48 | -56 | 50 |
| 7. | R postcentral gyrus | 47 | 8 | -28 | 60 |
| 8. | R paracentral lobule | 42 | 0 | -42 | 64 |
| 9. | L precentral gyrus | 41 | -14 | -28 | 58 |
| 10. | L thalamus | 40 | -14 | -12 | 0 |
| 11. | R middle temporal gyrus | 39 | 70 | -40 | -2 |
| 12. | L parahippocampal gyrus | 36 | -22 | -50 | 0 |
| 13. | R lingual gyrus | 34 | 8 | -94 | 2 |
| 14. | L posterior cingulate | 32 | 0 | -42 | 26 |
| 15. | L postcentral gyrus | 31 | -38 | -30 | 60 |
| 16. | L superior frontal gyrus | 30 | -20 | 54 | 14 |
| 17. | R cerebellum, anterior lobe | 27 | 24 | -56 | -28 |
| 18. | R cerebellum, posterior lobe | 27 | 16 | -60 | -18 |
| 19. | R precuneus | 27 | 20 | -60 | 50 |
| 20. | L precentral gyrus | 25 | -20 | -24 | 64 |
| 21. | R precentral gyrus | 24 | 32 | 0 | 30 |
| 22. | R lingual gyrus | 22 | 24 | -74 | -2 |
| 23. | L inferior parietal lobule | 22 | -48 | -32 | 34 |
| 24. | L posterior cingulate | 21 | -10 | -36 | 28 |
| 25. | brainstem | 20 | 0 | -24 | -20 |
| 26. | L thalamus | 20 | -2 | -16 | 0 |
| 27. | L inferior parietal lobule | 20 | -50 | -50 | 46 |
| **Decreased rCBF** | |  |  |  |  |
| 28. | L lateral orbitofrontal cortex | 134 | -26 | 18 | -18 |
| 29. | L superior temporal gyrus | 89 | -42 | 16 | -28 |
| 30. | R superior temporal gyrus | 56 | 30 | 16 | -36 |
